# Species Richness of Freshwater Fish Trophic Guilds Increases With Tropical River Discharge and Decreases With Variability

**DOI:** 10.1002/ece3.72343

**Published:** 2025-10-15

**Authors:** C. N. Perna, D. Sternberg, M. J. Kennard, O. J. Luiz, D. J. Irvine, D. Stratford, R. K. Kopf

**Affiliations:** ^1^ Research Institute for the Environment & Livelihoods, Faculty of Science and Technology Charles Darwin University Darwin Northern Territory Australia; ^2^ Burnett Mary Regional Group Bargara Queensland Australia; ^3^ Australian Rivers Institute Griffith University Nathan Queensland Australia; ^4^ CSIRO Environment Darwin Northern Territory Australia; ^5^ National Centre for Groundwater Research and Training Bedford Park South Australia Australia; ^6^ CSIRO Environment Black Mountain Australian Capital Territory Australia

**Keywords:** freshwater fish, natural flow regime, species area relationships, species richness of trophic guilds, trophic theory of island biogeography, tropical Australia

## Abstract

Species‐area relationships (SARs) are one of the most well‐established conservation biogeography patterns, and in rivers, habitat area is mediated by discharge. Species richness and river discharge have a well‐established positive relationship, but how discharge affects trophic diversity is less clear. Free‐flowing tropical river ecosystems are hotspots of global biodiversity, but they are under increasing threat from water resource developments which alter river discharge regimes. Here we investigate relationships between river discharge metrics and the species richness of freshwater fish trophic guilds in tropical rivers of northern Australia, using data collated from 40 catchments. We analyzed relationships between the species richness of freshwater fish trophic guilds and discharge metrics including mean annual discharge (Q), mean daily dry and wet season discharge, and the coefficient of variation (CVQ) of Q. Invertivores and omnivores were the most species‐rich trophic guilds. Our results show that the species richness of trophic guilds in north Australian freshwater fishes was correlated with multiple components of wet‐dry tropical river discharge regimes. The species richness of predators, invertivores, and herbivore‐detritivores increased with Q and wet season discharge, whereas omnivore and invertivore richness increased with dry season discharge. Increasing variability in discharge had a negative effect on the species richness of invertivores and omnivores, suggesting adverse effects of low discharge periods. We found no statistical support for the hypothesis that the slope of SARs increases with trophic level, as suggested by previous research. These findings suggest that decreases in wet and dry season discharge, or increases in flow variability due to water resource development or climate change, may result in the loss of trophic diversity from tropical rivers. Our results suggest that the conservation of both wet and dry season natural flow regimes in tropical rivers will be needed to protect freshwater fish trophic diversity.

## Graphical abstract

Contrary to the trophic theory of island biogeography the slope of predators does not increase with discharge when used to replace area. However, dry season discharge and variability in annual discharge are more strongly correlated with omnivore and invertivore guilds of freshwater fish than other discharge metrics. The credit is Fish images courtesy of the NESP Resilient Landscapes Hub, nesplandscapes.edu.au.

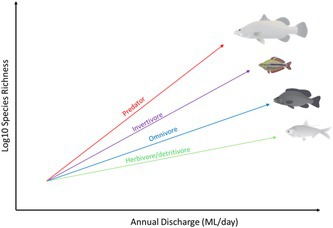

## 1 Introduction

Species‐area relationships (SARs) are among the most well‐established patterns in ecology and conservation biogeography and describe the positive relationship between species richness and habitat area (Macarthur and Wilson 1963, 1967). SARs have been tested and applied to a diverse range of ecosystems including islands (Macarthur and Wilson 1963, 1967), reefs (Stier et al. 2014), habitat patches (Brown and Dinsmore 1988), lakes (Gravel et al. 2011; Matias et al. 2017), and rivers (McGarvey 2014; Oberdorff et al. 1995). Despite limitations (He and Hubbell 2011), SARs remain widely used in conservation biogeography particularly for understanding biodiversity loss associated with habitat degradation on land (Rybicki and Hanski 2013) and in aquatic ecosystems (Xenopoulos et al. 2005; Xenopoulos and Lodge 2006; Tedesco et al. 2013).

In aquatic ecosystems, river basins are comparable to Macarthur and Wilson (1963, 1967) islands since geomorphological features act as barriers that constrain the movements of freshwater fishes and other aquatic animals. However, river habitat area is heterogeneous in space and time owing to dynamic flow discharge regimes, and thus is fundamentally different than terrestrial island habitats (Horwitz 1978; Poff 1997; Humphries et al. 2014). Global patterns of riverine fish species richness can be reliably predicted by mean annual discharge (Oberdorff et al. 1995), and the strength of the positive relationship can be improved by other measures of discharge (McGarvey 2014; Olden and Poff 2003; Poff 1997), including metrics associated with the timing, magnitude, frequency, and variation in discharge (Horwitz 1978; Oberdorff et al. 2011; McGarvey 2014). Other factors such as temperature and terrestrial productivity are correlated with species richness, but river discharge is considered an excellent predictor of the species richness of aquatic biota at the river basin scale (Poff 1997; Lamouroux et al. 2002; Kennard et al. 2010; Freeman et al. 2022). Despite its well‐supported role in predicting species richness, it is less clear how discharge relates to species richness within trophic guilds or other functional groups (Sternberg and Kennard 2013).

**FIGURE 1:**
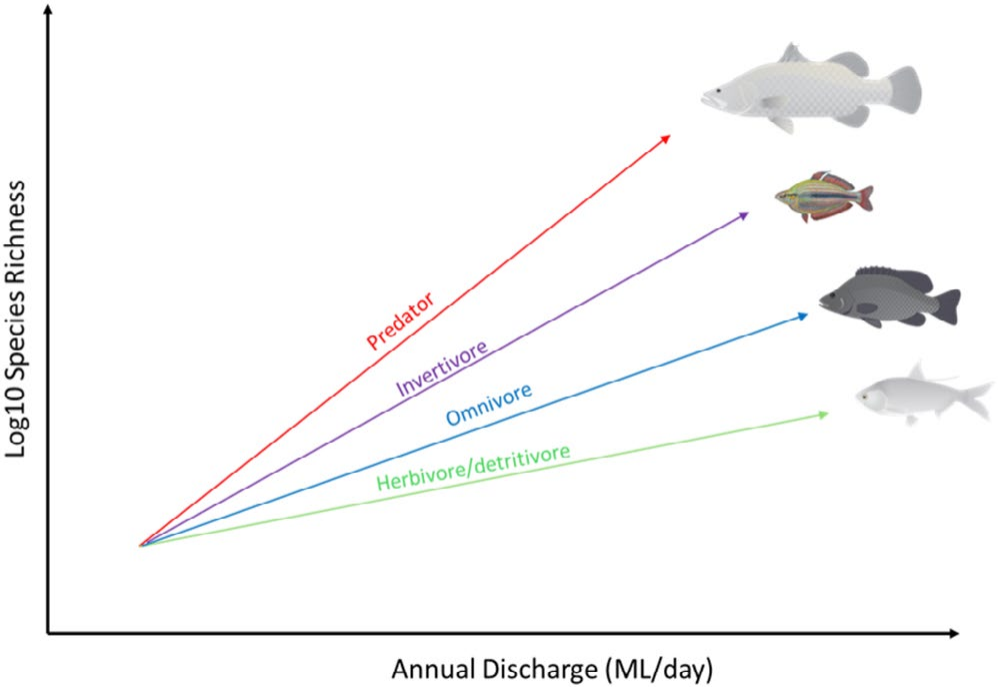
Conceptual model of hypotheses tested where predators have the steepest species area relationship (SAR) slopes and lower trophic level consumers have shallower slopes. Conceptual model adapted from Holt et al. (1999) and Holt (2010). The credit is Fish images courtesy of the NESP Resilient Landscapes Hub, nesplandscapes.edu.au.

Building upon Macarthur and Wilson (1967) theory of island biogeography, food web dynamics can improve predictions of species distributions (Gravel et al. 2011). Previous research suggests that the slope of SAR's may steepen (Holt et al. 1999; Holt 2010) with trophic position (Figure 1), whereby predators have the steepest slopes and lower consumers have shallower slopes. Predators are generally rarer than prey and more likely to go extinct, on small islands due to environmental stochasticity (Holt 2010). Additionally, higher trophic level consumers depend on the colonization and diversity of potential prey items lower in the food web (Holt 2010; Stortini et al. 2018). Predator diversity may therefore be more severely impacted by loss of habitat when compared to lower trophic level consumers (Roslin et al. 1991). However, relatively few studies have comprehensively tested whether the slope of SARs varies with trophic position. For example, trophic position may have little influence on SARs when top‐down interactions are important, communities are open with immigration, when consumers are generalist feeders, or when systems are far from equilibrium (Holt et al. 1999).

Tropical river fish communities are characterized by high trophic diversity and specialization (Winemiller et al. 2008). However, omnivory is widespread (Winemiller and Leslie 1992; Ceneviva‐Bastos et al. 2012), and food web dynamics might be shaped by both bottom‐up and top‐down (Douglas et al. 2005). Additionally, common large‐scale dispersal (Winemiller and Jepsen 1998) and high hydrological variability due to flood pulses (Jardine et al. 2015; Chea et al. 2020) may further shape SAR.

Free‐flowing rivers are some of the most biodiverse ecosystems on the planet (Grill et al. 2019), while at the same time species inhabiting these ecosystems are experiencing a higher rate of extinction than those in marine or terrestrial systems (He et al. 2022). Tropical rivers are under increasing pressure from water resource development (Toussaint et al. 2016; Winemiller et al. 2016), yet their impacts on functional and trophic diversity remain largely unknown. This paper investigates relationships between species richness of fish trophic guilds and river discharge in free‐flowing rivers of tropical Australia. We test whether the slope of species richness–river discharge relationships varies among trophic guilds (Figure 1). Further, we explore how these relationships differ among four discharge metrics: mean annual discharge, mean wet season discharge, mean dry season discharge, and annual discharge. We discuss the implications of these patterns for understanding the impact of water resource development and climate change on the trophic diversity of freshwater fishes in tropical rivers.

## 2 Materials and Methods

### 2.1 Fish Species and Trophic Guilds

We collated fish species richness and trophic guild data from Sternberg and Kennard (2013, 2014), and updated taxonomy and distribution data using Fishbase (2016), Atlas of Living Australia (https://www.ala.org.au/), and Pusey et al. (2017). This compilation resulted in 127 species that either reproduce in freshwater or diadromous species that spend a large amount of their life cycle in freshwater (Allen et al. 2002; Pusey, Arthington, and Kennard 2004). Occurrence data represented all described freshwater fishes known to occur in each of 55 catchments across three drainage regions of the tropical northern region of Australia: North‐East Coast, Gulf of Carpentaria, and Timor Sea (Figure S1; Table S1). Analyzed catchments are complete watersheds, extending from source to sea. These are delineated by elevation gradients and grouped in drainage basins which correspond to the marine regions into which the rivers flow, and they hold biogeographic significance. Small numbers of species may have gone undetected in some catchments, and new species are still being described. While this is the most comprehensive species richness database available for these rivers, there will be additions to the species list as new records are added and species described; however, these would unlikely alter the results of our models.

Our focal catchments occur within the tropics but vary in discharge from perennial discharge in the humid tropics of the North‐East Coast to seasonal or perennial groundwater discharge in the seasonal tropics and savannah of the Gulf of Carpentaria and Timor Sea basin areas (Duvert et al. 2022) (Figure S1, Table 1). The catchments include the highest diversity of freshwater fish within Australia, including 27, families, and 127 species (Allen et al. 2002; Pusey, Arthington, and Kennard 2004).

**TABLE 1:** Average annual discharge Q (ML/D) and coefficient of variation of Q for each of the three drainage basins for tropical northern Australia. Drainage basins are defined by hydrologic, geographic, and biogeographical criteria.

Drainage basin	Mean Q (ML/D) ± SE	CV of Q
North‐East Coast (*n* = 13)	41,6613 ± 1437	0.10
Gulf of Carpentaria (*n* = 12)	5328 ± 1460	0.95
Timor (*n* = 15)	101,311 ± 1824	0.07

The trophic guilds for all listed species in each catchment were determined using a variety of sources, including Allen et al. (2002); Pusey, Kennard, and Arthington (2004); Sternberg and Kennard (2014) and Fishbase (2016). Species were grouped in four trophic guilds: (1) Herbivores/Detritivores: fish that consume algae, plants, and detrital material; (2) Omnivores: fish with a broad range of food sources consumed in variable proportions, which can include plankton, algae, macrophytes, and invertebrates (terrestrial and aquatic); (3) Invertivores: fish that consume terrestrial and aquatic invertebrates and crustaceans, and sometimes fish prey; (4) Predators: fish that largely consume fish and aquatic crustaceans, and to a lesser extent terrestrial and aquatic invertebrates (Allen et al. 2002; Pusey, Arthington, and Kennard 2004; Sternberg and Kennard 2014; Fishbase 2016).

### 2.2 River Discharge Data

Surface water data were obtained from the Global Runoff Data Centre (GRDC) (2023), the Australian Bureau of Meteorology (BOM) (2023), the Queensland Government (2023), the NT Government (2023), and Geoscience Australia (2004) across three drainage basins: North‐East Coast, Gulf of Carpentaria, and Timor Sea. See Figure S1 for the location of hydrographic logging stations. Where available (i.e., 40 out of 55 catchments), data were used from the most downstream gauging station. Raw data that were available at a sub‐daily timestep were converted to daily mean discharge. Linear interpolation was used to fill gaps of 3 days or less. Larger gaps were not filled, and all data management steps, including daily average and percentile calculations, were performed with the Pandas (Mckinney 2010) and Numpy libraries in Python.

Owing to the absence of hydrological gauging stations and reliable discharge data in 15 catchments, statistical analyses were limited to 40 out of the 55 catchments in the study region. Each hydrograph was analyzed by water year (starting September) to allow the calculations to capture a single wet season (all calculations made in Python using Numpy and Panda's libraries) and beginning in April for the dry season. Discharge variables explored initially included standard deviation, quartiles of discharge, and the total number of zero discharge days across the entire record (Table S2). However, given the collinearity among many discharge metrics, we reduced the statistical analyses to interactions with four discharge variables: mean annual discharge = Q (ML/d); mean wet season discharge (Wet; ML/d); mean dry season discharge (Dry; ML/d); and the coefficient of variation in mean annual discharge (CVQ). These variables were log_10_ transformed. Summaries of the catchments assessed, the number of water years contained within each dataset, and the percentage of data gaps are provided in Tables S3–S5. These calculations were performed on the data records after the missing records were removed.

### 2.3 Statistical Analysis

To evaluate whether there were differences in fish species richness among trophic guilds and whether richness patterns were correlated with discharge metrics, we fitted Generalized Linear Mixed Effects Models (GLMM) with a Gaussian family distribution, using the ‘lme4’ Package (Bates et al. 2015) in R studio (version 2023.12.1 Build 402). The species richness in each trophic guild was collated and combined with catchment river discharge data (Table S6). We tested 20 models (Table 2) to evaluate the effects of the four discharge predictor variables (Q; Wet season discharge; Dry season discharge; CVQ) on total species richness and species richness of each trophic guild. The models were evaluated based on slope estimates, Marginal *R* ^2^ and *p* values using the *lme4* R package (Bates et al. 2015). Akaike Information Criterion (AIC) was used to determine the best discharge predictor for each trophic guild but was not be used to compare across trophic guilds owing to different response variables. Catchments were grouped according to drainage basins and set as random effects to reduce spatial autocorrelation (Table 2). To test for pair‐wise differences in the slopes between trophic guilds, we used the *emtrends* function from the *emmeans* R package (Lenth et al. 2023).

**TABLE 2:** Model descriptions and results for GLMM analysis. There was no variance in random effects; therefore, no Conditional *R* ^2^. Significant models are in bold.

Model	df	AIC	Marginal *R* ^2^	*p*	Slope	SE
**Predator ~ Wet + (1| basin)**	**36**	**−9.08**	**0.14**	**0.02**	**0.06**	**0.02**
**Invertivore ~ Wet + (1| basin)**	**36**	**14.53**	**0.14**	**0.02**	**0.08**	**0.03**
Omnivore ~ Wet + (1| basin)	36	15.13	0.09	0.06	0.06	0.03
**Herbivore/Detritivore ~ Wet + (1| basin)**	**36**	**8.23**	**0.14**	**0.02**	**0.07**	**0.03**
Predator ~ Dry + (1| basin)	36	−3.93	0.04	0.19	0.02	0.02
**Invertivore ~ Dry + (1| basin)**	**36**	**9.86**	**0.25**	**0.001**	**0.07**	**0.02**
**Omnivore ~ Dry + (1| basin)**	**36**	**12.38**	**0.17**	**0.01**	**0.05**	**0.02**
Herbivore/Detritivore ~ Dry + (1| basin)	36	11.29	0.09	0.06	0.04	0.02
Predator ~ CVQ + (1| basin)	36	−6.84	0.03	0.25	−0.09	0.08
**Invertivore ~ CVQ + (1| basin)**	**36**	**7.89**	**0.23**	**0.001**	**−0.33**	**0.10**
**Omnivore ~ CVQ + (1| basin)**	**36**	**9.52**	**0.16**	**0.01**	**−0.27**	**0.10**
Herbivore/Detritivore ~ CVQ + (1| basin)	36	11.49	< 0.00	0.76	0.03	0.10

## 3 Results

Species richness was the highest for invertivores (*n* = 54), followed by omnivores (*n* = 36), predators (*n* = 21), and herbivore‐detritivores (*n* = 16) (Figure S2) for all the combined catchments. The total species richness significantly increased with Q, wet and dry season discharge metrics but declined with CVQ (Figure S3, Table S7). There were significant differences in the slopes of trophic guild species richness–discharge relationships (Table 2), but the results did not follow the hypothesized (Figure 1) pattern of increasing slope with trophic position for any of the flow variables measured.

**FIGURE 2:**
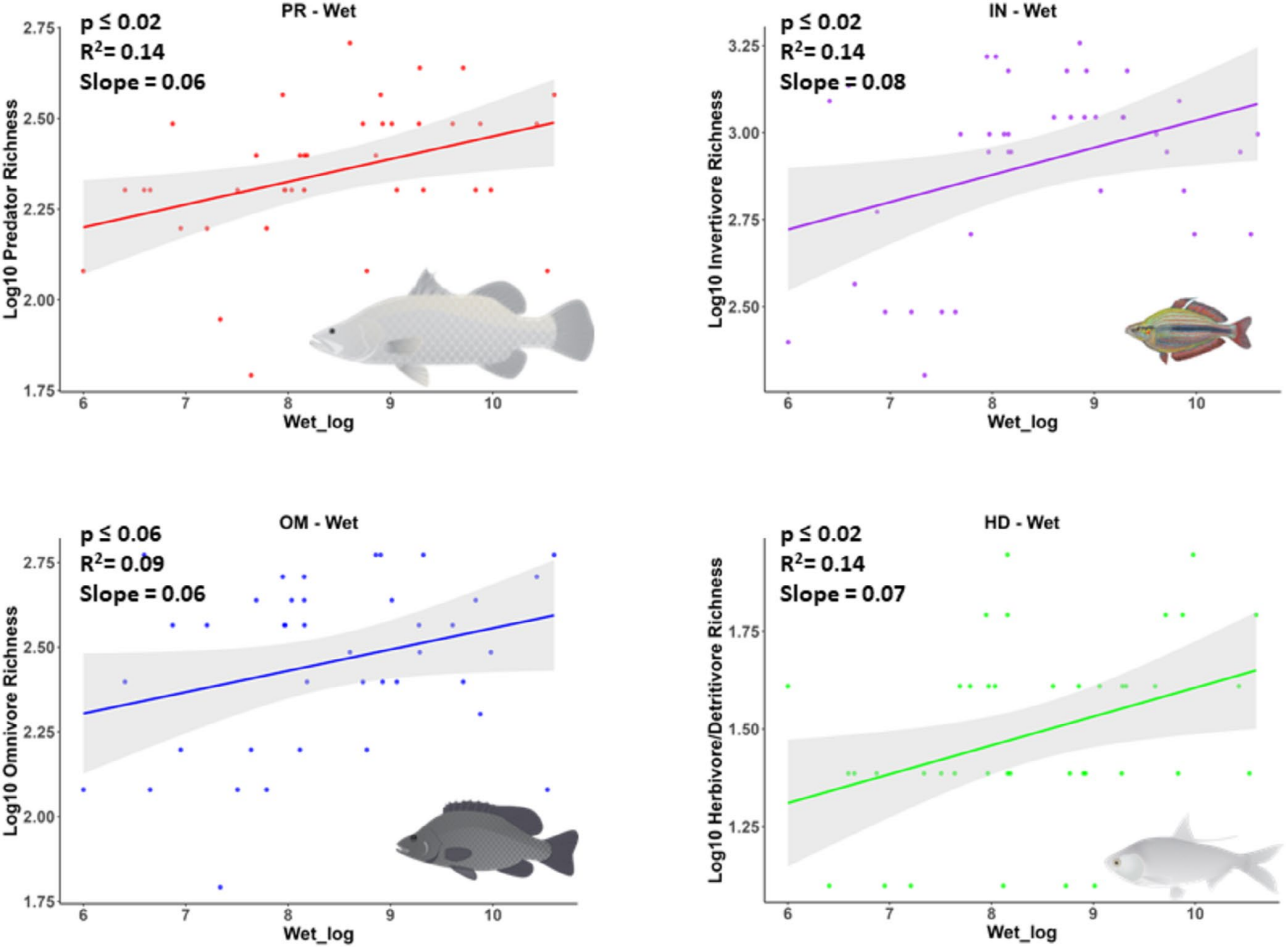
Fitted linear models, including 95% confidence intervals, showing species richness of each trophic guild in relation to mean wet season discharge (Wet_log = ML/d). All data are log10 transformed. Each datapoint represents the number of species in a catchment. The credit is Fish images courtesy of the NESP Resilient Landscapes Hub, nesplandscapes.edu.au.

**FIGURE 3:**
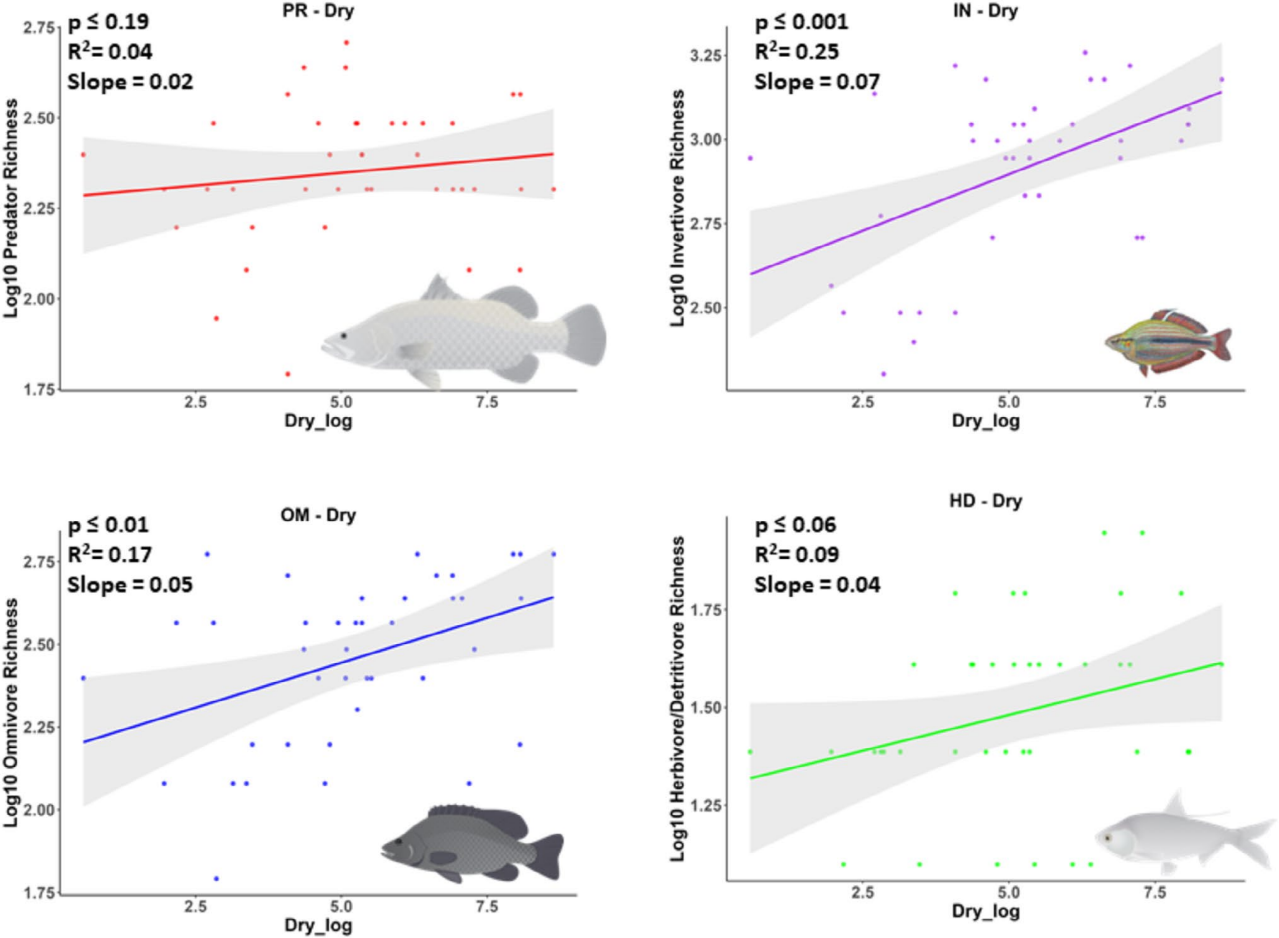
Fitted liner models, including 95% confidence intervals in the shaded area, for species richness of each trophic guild for mean dry season discharge (Dry_log = ML/d). All data are log10 transformed. Each datapoint represents the number of species in a catchment. The credit is Fish images courtesy of the NESP Resilient Landscapes Hub, nesplandscapes.edu.au.

**FIGURE 4:**
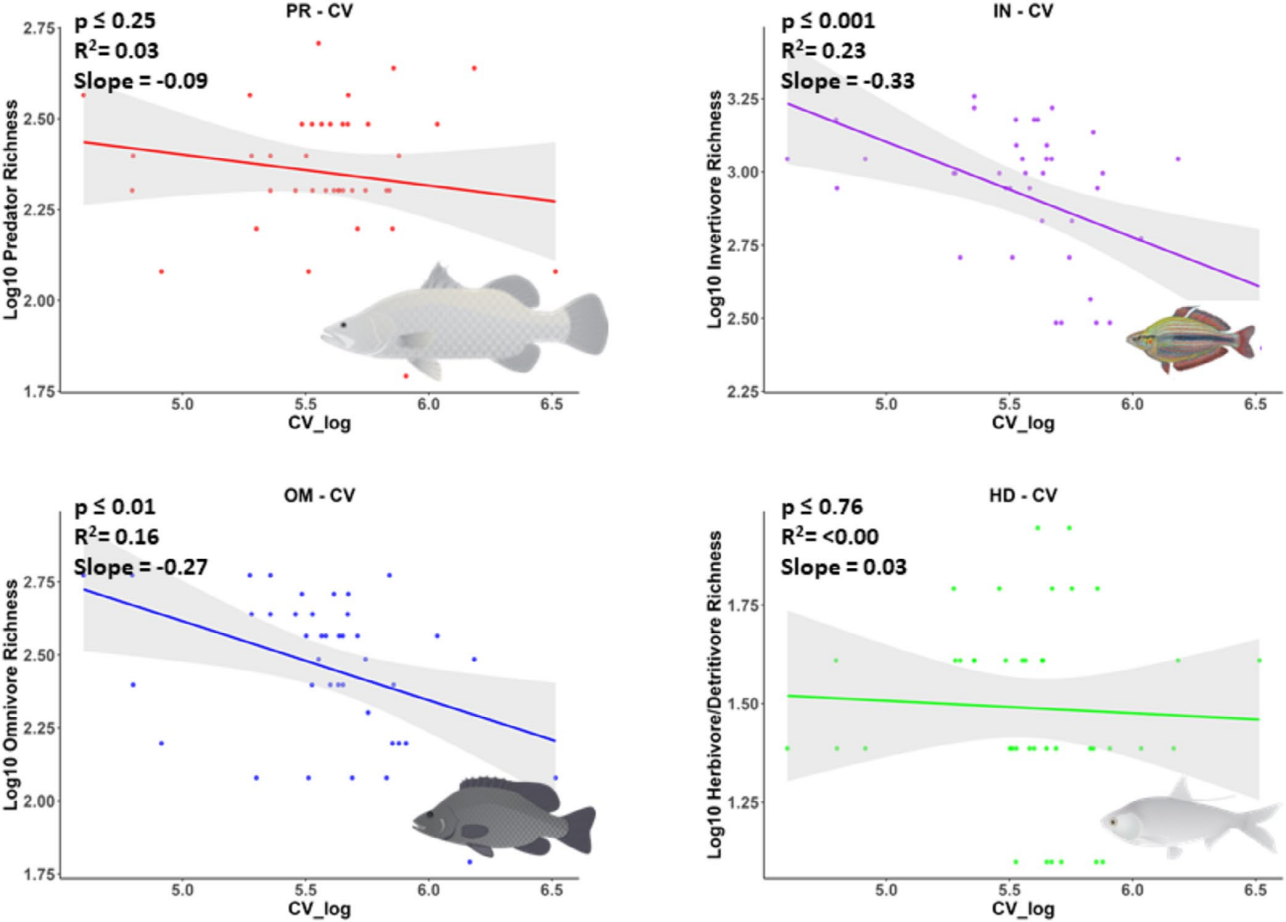
Fitted linear models, including 95% confidence intervals, showing the species richness of each trophic guild in relation to the mean coefficient of variation of mean annual discharge (CV_log = ML/d). All data are log10 transformed. Each datapoint represents the number of species in a catchment. The credit is Fish images courtesy of the NESP Resilient Landscapes Hub, nesplandscapes.edu.au.

The species richness of predators, invertivores, and herbivore/detritivores increased with mean daily wet season discharge (Figure 2), but pair‐wise comparisons showed that slopes were not significantly different (Table S8). A statistically significant relationship was not detected for omnivore richness in relation to mean daily wet season discharge (Table 2), but omnivore and invertivore richness increased significantly in relation to mean daily dry season discharge (Figure 3) and significantly decreased in relation to CVQ (Figure 4). Pair‐wise comparisons suggested that the slopes of species richness of invertivores and omnivores in relation to flow metrics were not significantly different (Table S8). Statistically significant relationships were not detected for herbivore‐detritivore or predator richness in relation to mean daily dry season discharge or CVQ (Figures 3 and 4).

Consistent with the differences observed among the slopes for different trophic guilds, the Akaike Information Criterion (AIC) suggested that the most parsimonious models explaining predator and herbivore–detritivore species richness were wet season discharge (Table 2). The most parsimonious model explaining invertivore and omnivore richness included CVQ, while dry season and wet season discharge models, respectively, provided less parsimonious models.

## 4 Discussion

This study quantifies the species richness–discharge relationships across trophic guilds in tropical free‐flowing rivers. Our results show that the species richness of trophic guilds in northern Australian freshwater fishes was correlated with multiple components of the wet–dry tropical river discharge regime. The species richness of predators, invertivores, and herbivore‐detritivores increased with wet season discharge. Omnivore and invertivore richness increased with dry season discharge, but the slopes were not significantly different. Importantly, increasing variability in mean annual river discharge (CVQ) had significant negative effects on the species richness of invertivores and omnivores, suggesting adverse effects of flow variability. However, in contrast to expectations based on ecological theory (Holt et al. 1999; Holt 2010), these slopes were not significantly different among different trophic guilds.

While wet‐dry discharge regimes were significant factors affecting the species richness of fish trophic guilds, other drivers related to phylogeny, morphological traits, biogeography, and climate play important roles in supporting the trophic species richness of tropical river fishes (Luiz et al. 2025). Discharge also does not account for habitat heterogeneity directly, but it is often correlated with it. Greater discharge can increase habitat complexity, which in turn can stabilize food webs and suppress the SARs slope increases with trophic level (Holt 2010). Furthermore, the widespread occurrence of omnivory in tropical freshwater fish (Ceneviva‐Bastos et al. 2012), the importance of top‐down controls within food webs (Douglas et al. 2005), and the prevalence of large‐scale dispersal and diadromy (Pusey, Arthington, and Kennard 2004) all contribute to species‐area dynamics. In addition, the tropical river discharge in Australia is highly variable (Perna and Pearson 2008; Jardine et al. 2015), and the predictions of Holt (2010) and Holt et al. (1999) may have limited applicability in these arhythmic tropical rivers of Australia. The variability created by the arhythmic nature of Australian wet season floods supports more omnivory and opportunism in fish species (Jardine et al. 2015) as opposed to those in more rhythmic rivers in the Amazon or Mekong supporting higher prevalence of trophic specialization (Winemiller 2004).

The freshwater fish fauna of tropical Australia is relatively depauperate in comparison to other tropical river systems (Pusey, Kennard, and Arthington 2004; Das et al. 2012; Winemiller et al. 2015; Chea et al. 2020). Our results support hypotheses suggesting that lower river discharge and higher annual flow variability (Pusey et al. 2000; Jardine et al. 2015) play key roles in explaining species richness (Bunn and Arthington 2002; Oberdorff et al. 2011). The positive correlations between omnivore and invertivore richness and dry season discharge are consistent with general patterns in highly variable lotic environments (Wantzen et al. 2002), where dietary flexibility allows species to persist despite fluctuations in resource availability.

The observed increase in predator species richness with wet season discharge highlights the importance of flooding for this guild (Roberts et al. 2019). Wet season flows facilitate the migration of predators to prey‐rich wetland habitats (Winemiller and Jepsen 1998; Roberts et al. 2023), and can increase recruitment to the adult population (Leahy and Robins 2021; Roberts et al. 2021) through increased prey density and access to predator refuges. Understanding the relationship between discharge and predator species richness is key for biodiversity and fisheries management. For example, this knowledge can advise water resource planning and climate change mitigation strategy, both of which can reduce wet season flooding events that connect predators to prey and habitats (O'Mara et al. 2025), potentially affecting the predator species richness in impacted rivers.

McGarvey (2014) found that fish species richness–discharge relationships in the Pacific Northwest were the strongest when using low‐discharge (winter) metrics. Similarly, our results suggest that low‐discharge conditions during the dry season in tropical rivers strongly influence species richness of some trophic guilds. Periods of low discharge reduce the extent of physical habitat, change water quality and habitat condition, and reduce allochthonous resource availability and river connectivity, all of which can impact community structure and trophic dynamics (Rolls et al. 2012). These effects may be reflected in the significant slopes for invertivores and omnivores that rely on allochthonous materials during the dry season. In contrast, herbivores showed little to no response, probably because of their dependency on autochthonous food sources (Pusey et al. 2021).

The species richness of all trophic guilds declined with increasing discharge variability, suggesting that high annual variation in discharge is an important driver of fish extirpation dynamics in tropical rivers. However, predator and herbivore/detritivore richness were not correlated significantly with discharge variability, further suggesting that predators in these rivers may be feeding at several trophic levels, depending on water levels and prey availability, and are less susceptible to bottom‐up trophic effects. Alternatively, large predators and some herbivorous fishes in northern Australia are diadromous or have a high salinity tolerance (Luiz et al. 2025), which may allow them to escape the adverse effects of high river flow variability. In contrast, many small‐bodied invertivores and omnivores are freshwater obligate species that contribute significantly to regional species richness (Sternberg and Kennard 2013; Griffiths et al. 2014; Luiz et al. 2025). Their limited mobility, small body sizes, and low salinity tolerance might make them more vulnerable but confine them to freshwater environments and flow variability. These traits can amplify SAR slopes, reflecting their higher dependence on stable environments. On the other hand, predators often exhibit trophic flexibility, large body size, higher mobility, and diadromous behavior, buffering them against flow fluctuations and flattening their SAR slopes.

Tropical rivers worldwide are undergoing expansion in water resource development (Winemiller et al. 2016; He et al. 2024), with several plans proposed in northern Australia to extract water primarily for agriculture (Australian Government 2015). These developments are expected to alter discharge regimes and affect biodiversity (King et al. 2024; Humphries et al. 2024). Unlike most temperate river systems that have been extensively developed and studied (Barmuta 2003), tropical Australian rivers remain mostly free‐flowing (Warfe et al. 2011) and have higher biodiversity (Pusey, Kennard, and Arthington 2004), but face increasing threats from developments and climate change. Despite the important potential impacts of discharge alteration, tropical rivers have received much less research attention than temperate ecosystems. Our results show consistent positive correlations between fish species richness and both wet and dry season discharge across all trophic guilds, suggesting that discharge reductions in either season may result in species richness losses in all guilds. Invertivore and omnivore guilds showed stronger correlations with dry season discharge, making them particularly vulnerable to dry season reductions from water extraction or climate change.

This vulnerability of tropical freshwater fish communities to dry season discharge alteration has been recognized in Australia (Arthington and Pusey 2003; Morrongiello et al. 2011; King et al. 2024) and other tropical regions (Jorgensen et al. 2012). Our results extend these findings to include losses across all trophic levels and demonstrate sensitivity to wet season discharge, consistent with previous work highlighting the importance of annual flooding and connectivity with adjacent floodplains (Pettit et al. 2017). These flood events support food web dynamics across all trophic guilds, particularly benefiting predators, but are increasingly threatened by water resource developments such as wet season floodplain harvest and off‐channel water storages (Petheram et al. 2018). For example, the Adelaide River Off‐Channel Water Storage Scheme (AROWS) plans to extract water from the free‐flowing Adelaide River during wet season flooding. Our results suggest that reduced wet season discharge may disproportionally impact predators, herbivores/detritivores, and invertivores.

To support freshwater fish species richness in undeveloped and biodiverse tropical rivers, water resource developments should incorporate discharge‐ecology relationships in conservation planning to minimize biodiversity impacts from proposed developments and climate change. Climate change impacts on tropical river discharge in northern Australia remain uncertain due to contrasting rainfall predictions that may increase or decrease river discharge (Morrongiello et al. 2011). While there is little evidence that drought frequency or intensity will increase in northern Australia's monsoonal regions (CSIRO and The Bureau of Meteorology 2020), dry seasons are expected to be longer, and more extreme floods with increased variability are anticipated (Morrongiello et al. 2011). Our results suggest that increasing discharge variability, primarily from annual wet season variation, would contribute to losses in freshwater species richness of all trophic guilds. Since tropical rivers of northern Australia naturally have among the highest discharge variability (Jardine et al. 2015), human‐driven increases through climate change or water resource development may adversely affect species richness. However, if climate change produces higher rainfall and larger, more consistent floods, this could positively impact fish trophic species richness in tropical rivers (Wang et al. 2024). Further research is needed to improve climate projections of river discharge, drought, and flood patterns and their biodiversity effects in tropical rivers.

## Author Contributions

**C. N. Perna:** conceptualization (lead), formal analysis (lead), methodology (lead), writing – original draft (lead), writing – review and editing (lead). **D. Sternberg:** resources (supporting), writing – review and editing (supporting). **M. J. Kennard:** resources (supporting), writing – review and editing (supporting). **O. J. Luiz:** data curation (supporting), formal analysis (supporting), methodology (supporting), supervision (supporting), writing – review and editing (supporting). **D. J. Irvine:** data curation (supporting), formal analysis (supporting), writing – review and editing (supporting). **D. Stratford:** supervision (supporting), writing – review and editing (supporting). **R. K. Kopf:** conceptualization (supporting), formal analysis (supporting), methodology (supporting), supervision (supporting).

## Conflicts of Interest

The authors declare no conflicts of interest.

## Supporting information


**Table S1:** Fish species list including trophic guild and occurrence across 55 catchments.
**Table S2:** Discharge variables tested by total species richness and species richness of trophic guilds. Discharge variables were highly correlated reducing the number of models used to those with significant results (in bold).
**Table S3:** Description of river hydrograph data from the Tanami‐Timor Sea Coast (TTS) utilized in study. Sources are as follows; GRDC is the Global Runoff Data Centre, BoM is the Bureau of Meteorology Water Data Online, NTG is the Northern Territory Government Aquatic Informatics site. Note, % complete includes filling minor gaps of 3 days or less using linear interpolation. Long term values of all water years with 90% or more complete records for mean flow (μQ), coefficient of variation of flow (CV_Q_), no flow days and the mean number of days per water year where flows were between historical 30th, 70th and 90th percentiles of flow.
**Table S4:** Description of river hydrograph data from the Carpentaria Coast (CC) utilized in study. Sources are as follows; GRDC is the Global Runoff Data Centre, BoM is the Bureau of Meteorology Water Data Online, NTG is the Northern Territory Government Aquatic Informatics site. Note, % complete includes filling minor gaps of 3 days or less using linear interpolation. Long term values of all water years with 90% or more complete records for mean flow (μQ), coefficient of variation of flow (CV_Q_), no flow days and the mean number of days per water year where flows were between historical 30th, 70th and 90th percentiles of flow.
**Table S5:** Description of river hydrograph data from the North East Coast (NEC) utilized in study. Sources are as follows; GRDC is the Global Runoff Data Centre, BoM is the Bureau of Meteorology Water Data Online, NTG is the Northern Territory Government Aquatic Informatics site. Note, % complete includes filling minor gaps of 3 days or less using linear interpolation. Long term values of all water years with 90% or more complete records for mean flow (μQ), coefficient of variation of flow (CV_Q_), no flow days and the mean number of days per water year where flows were between historical 30th, 70th and 90th percentiles of flow.
**Table S6:** Total species richness and species richness of trophic guilds by catchment including discharge metrics.
**Table S7:** Model descriptions and results for GLMM analysis of total species richness across all discharge predictors. There was no variance in random effects therefore no Conditional *R* ^2^.
**Table S8:** Pairwise contrast table for the four trophic guilds across four flow predictors.
**Figure S1:** Locations of the hydrologic gauging stations, drainage divisions and catchment boundaries used to assess fish species richness in tropical Australia.
**Figure S2:**. Box plot of trophic guild richness of freshwater fishes in tropical Australia, and coefficient estimates ±95% confidence intervals.
**Figure S3:** Fitted liner models slopes, including 95% confidence intervals in the shaded area, for total species richness of mean annual discharge Q (ML/d), Wet season discharge (ML/d), Dry season discharge (ML/d) and Coefficient of Variation for mean annual discharge (CVQ). All data was is log10 transformed. Each datapoint represents the number of species in a catchment.

## Data Availability

All data are publicly available on the Dryad archiving website at https://doi.org/10.5061/dryad.zkh1893nr.
