# Supplementary material for: Species Richness of Freshwater Fish Trophic Guilds Increases With Tropical River Discharge and Decreases With Variability
Source: Ecol Evol. 2025 Oct 15;15(10):e72343. doi: 10.1002/ece3.72343 (PMC12527586; doi:10.1002/ece3.72343)
Supplement: Supplementary file 1 — Table S1: Fish species list including trophic guild and occurrence across 55 catchments. Table S2: Discharge variables tested by total species richness and species richness of trophic guilds. Discharge variables were highly correlated reducing the number of models used to those with significant results (in bold). Table S3: Description of river hydrograph data from the Tanami‐Timor Sea Coast (TTS) utilized in study. Sources are as follows; GRDC is the Global Runoff Data Centre, BoM is the Bureau of Meteorology Water Data Online, NTG is the Northern Territory Government Aquatic Informatics site. Note, % complete includes filling minor gaps of 3 days or less using linear interpolation. Long term values of all water years with 90% or more complete records for mean flow (μQ), coefficient of variation of flow (CVQ), no flow days and the mean number of days per water year where flows were between historical 30th, 70th and 90th percentiles of flow. Table S4: Description of river hydrograph data from the Carpentaria Coast (CC) utilized in study. Sources are as follows; GRDC is the Global Runoff Data Centre, BoM is the Bureau of Meteorology Water Data Online, NTG is the Northern Territory Government Aquatic Informatics site. Note, % complete includes filling minor gaps of 3 days or less using linear interpolation. Long term values of all water years with 90% or more complete records for mean flow (μQ), coefficient of variation of flow (CVQ), no flow days and the mean number of days per water year where flows were between historical 30th, 70th and 90th percentiles of flow. Table S5: Description of river hydrograph data from the North East Coast (NEC) utilized in study. Sources are as follows; GRDC is the Global Runoff Data Centre, BoM is the Bureau of Meteorology Water Data Online, NTG is the Northern Territory Government Aquatic Informatics site. Note, % complete includes filling minor gaps of 3 days or less using linear interpolation. Long term values of all water years with [file ECE3-15-e72343-s001.docx]

**Supplemental table 1.** Fish species list including trophic guild and occurrence across 55 catchments.

| **Family** | **Species** | **Trophic Guild** | **Occurrence** |
| --- | --- | --- | --- |
| Anguillidae | *Anguilla bicolor* | 4 | 3 |
| Anguillidae | *A. obscura* | 4 | 13 |
| Anguillidae | *A. reinhardtii* | 4 | 14 |
| Apogonidae | *Glossamia aprion* | 4 | 40 |
| Ariidae | *Cinetodus froggatti* | 3 | 4 |
| Ariidae | *Neoarius berneyi* | 2 | 12 |
| Ariidae | *N. graeffei* | 2 | 29 |
| Ariidae | *N. leptaspis* | 4 | 24 |
| Ariidae | *N. midgleyi* | 4 | 7 |
| Ariidae | *N. paucus* | 4 | 15 |
| Atherinidae | *Craterocephalus lentigenosus* | 2 | 2 |
| Atherinidae | *C. marianae* | 2 | 4 |
| Atherinidae | *C. stercusmuscarum* | 2 | 35 |
| Atherinidae | *C. stramineus* | 2 | 5 |
| Belonidae | *Strongylura krefftii* | 4 | 39 |
| Centropomidae | *Lates calcarifer* | 4 | 40 |
| Chandidae | *Ambassis agassizii* | 3 | 7 |
| Chandidae | *A. agrammus* | 3 | 37 |
| Chandidae | *A. elongatus* | 3 | 6 |
| Chandidae | *A. interrupta* | 3 | 21 |
| Chandidae | *A. macleayi* | 3 | 25 |
| Chandidae | *A. miops* | 3 | 8 |
| Chandidae | *A. sp.* | 3 | 24 |
| Chandidae | *Denaiusa australis* | 3 | 24 |
| Chandidae | *Parambassis gulliveri* | 4 | 4 |
| Clupeidae | *Nematalosa erebi* | 1 | 40 |
| Cynoglossidae | *Cynoglossus heterolepis* | 4 | 3 |
| Eleotridae | *Bostrichthys zonatus* | 3 | 13 |
| Eleotridae | *Bunaka gyrinoides* | 3 | 10 |
| Eleotridae | *Eleotris fusca* | 3 | 9 |
| Eleotridae | *E. melanosoma* | 3 | 7 |
| Eleotridae | *Giurus aporocephalus* | 2 | 15 |
| Eleotridae | *Hypseleotris bucephala* | 2 | 3 |
| Eleotridae | *H. burrawayi* | 2 | 1 |
| Eleotridae | *H. compressa* | 2 | 40 |
| Eleotridae | *H. galli* | 3 | 1 |
| Eleotridae | *H. kimberleyensis* | 3 | 2 |
| Eleotridae | *H. klunzingeri* | 3 | 1 |
| Eleotridae | *Mogurnda adspersa* | 3 | 11 |
| Eleotridae | *M. mogurnda* | 3 | 31 |
| Eleotridae | *M. oligolepis* | 3 | 3 |
| Eleotridae | *Ophiocara porocephala* | 3 | 10 |
| Eleotridae | *Oxyeleotris aruensis* | 3 | 7 |
| Eleotridae | *O. fimbriata* | 3 | 3 |
| Eleotridae | *O. lineolatus* | 4 | 35 |
| Eleotridae | *O. nullipora* | 3 | 21 |
| Eleotridae | *O. selheimi* | 4 | 31 |
| Engraulidae | *Thryssa scratchleyi* | 4 | 6 |
| Gobiidae | *Awaous acritosus* | 2 | 11 |
| Gobiidae | *Chlamydogobius ranunculus* | 3 | 9 |
| Gobiidae | *Glossogobius aureus* | 3 | 24 |
| Gobiidae | *G. celebius* | 3 | 10 |
| Gobiidae | *G. concavifrons* | 3 | 3 |
| Gobiidae | *G. giuris* | 3 | 37 |
| Gobiidae | *Glossogobius sp. 2* | 3 | 23 |
| Gobiidae | *Glossogobius sp. 3* | 3 | 8 |
| Gobiidae | *Mugilogobius notospilus* | 3 | 4 |
| Gobiidae | *Philypnogon grandiceps* | 3 | 1 |
| Gobiidae | *Psammogobius biocellatus* | 3 | 7 |
| Gobiidae | *Redigobius chryosoma* | 3 | 1 |
| Gobiidae | *R. bikolanus* | 3 | 13 |
| Gobiidae | *Schismatogobius sp.* | 3 | 5 |
| Gobiidae | *Sicyopterus lagocephalus* | 1 | 2 |
| Gobiidae | *Stiphodon allen* | 1 | 2 |
| Hemiramphidae | *Arramphus sclerolepis* | 1 | 32 |
| Hemiramphidae | *Zenarchopterus buffonis* | 2 | 13 |
| Hemiramphidae | *Z. caudovittatus* | 2 | 3 |
| Hemiramphidae | *Z. dispar* | 2 | 4 |
| Hemiramphidae | *Z. novaeguineae* | 2 | 10 |
| Kuhliidae | *Kuhlia marginata* | 2 | 4 |
| Kuhliidae | *K. rupestris* | 2 | 13 |
| Kurtidae | *Kurtus gulliveri* | 4 | 10 |
| Megalopidae | *Megalops cyprinoides* | 4 | 40 |
| Melanotaeniidae | *Cairnsichthys rhombosomoides* | 2 | 3 |
| Melanotaeniidae | *Iriatherina werneri* | 2 | 10 |
| Melanotaeniidae | *Melanotaenia australis* | 3 | 16 |
| Melanotaeniidae | *M. eachamensis* | 2 | 3 |
| Melanotaeniidae | *M. exquisita* | 2 | 5 |
| Melanotaeniidae | *M. maccullochi* | 2 | 11 |
| Melanotaeniidae | *M. nigrans* | 2 | 18 |
| Melanotaeniidae | *M. pygmaea* | 2 | 1 |
| Melanotaeniidae | *M. splendida* | 2 | 34 |
| Melanotaeniidae | *M. trifasciata* | 2 | 17 |
| Melanotaeniidae | *M. utcheensis* | 2 | 1 |
| Mugilidae | *Liza ordensis* | 1 | 19 |
| Mugilidae | *Mugil cephalus* | 1 | 9 |
| Osteoglossidae | *Scleropages jardinii* | 4 | 18 |
| Plotosidae | *Anodontoglanis dahli* | 3 | 22 |
| Plotosidae | *Neosilurus ater* | 3 | 38 |
| Plotosidae | *N. brevidorsalis* | 3 | 3 |
| Plotosidae | *N. hyrtlii* | 3 | 39 |
| Plotosidae | *N. mollespiculum* | 2 | 1 |
| Plotosidae | *N. pseuodospinosus* | 3 | 5 |
| Plotosidae | *Porochilus obbesi* | 3 | 10 |
| Plotosidae | *P. rendahli* | 3 | 35 |
| Plotosidae | *Tandanus tropicanus* | 3 | 6 |
| Pseudomugilidae | *Pseudomugil gertrudae* | 3 | 23 |
| Pseudomugilidae | *P. signifer* | 3 | 12 |
| Pseudomugilidae | *P. tenellus* | 2 | 13 |
| Scatophagidae | *Scatophagus argus* | 1 | 3 |
| Scatophagidae | *Selenotoca multifasciata* | 1 | 3 |
| Scorpaenidae | *Notesthes robusta* | 4 | 9 |
| Soleidae | *Aseraggodes klunzingeri* | 4 | 3 |
| Soleidae | *Brachirus salinarum* | 3 | 6 |
| Soleidae | *B. selheimi* | 3 | 7 |
| Soleidae | *Leptachirus triramus* | 3 | 3 |
| Synbranchidae | *Ophisternon gutturale* | 4 | 12 |
| Synbranchidae | *Ophisternon sp.* | 4 | 21 |
| Terapontidae | *Amniataba percoides* | 2 | 37 |
| Terapontidae | *Hannia greenwayi* | 2 | 2 |
| Terapontidae | *Hephaestus carbo* | 3 | 12 |
| Terapontidae | *Hephaestus fuliginosus* | 2 | 29 |
| Terapontidae | *H. jenkinsi* | 2 | 5 |
| Terapontidae | *H. tulliensis* | 2 | 5 |
| Terapontidae | *Leiopotherapon unicolor* | 2 | 40 |
| Terapontidae | *Pingalla gilberti* | 1 | 2 |
| Terapontidae | *P. lorentzi* | 1 | 3 |
| Terapontidae | *P. midgleyi* | 1 | 3 |
| Terapontidae | *Scortum neili* | 1 | 1 |
| Terapontidae | *S. ogilbyi* | 1 | 9 |
| Terapontidae | *S. parviceps* | 1 | 1 |
| Terapontidae | *Syncomistes butleri* | 1 | 12 |
| Terapontidae | *S. trigonicus* | 1 | 1 |
| Terapontidae | *Variichthys lacustris* | 2 | 1 |
| Toxotidae | *Toxotes chatereus* | 3 | 40 |
| Toxotidae | *T. kimberleyensis* | 3 | 3 |
| Toxotidae | *T. lorentzi* | 3 | 7 |

**Table 2.** Discharge variables tested by total species richness and species richness of trophic guilds. Discharge variables were highly correlated reducing the number of models used to those with significant results (in bold).

| **Discharge Variable Annual** | **Discharge variables (Per season wet and dry)** |
| --- | --- |
| **Annual mean discharge* (Q)** | Maximum discharge |
| **Coefficient of variation*** | Standard deviation |
| Standard deviation | Total number of zero discharge days |
| Quartiles of discharge | Days below 10^th^ percentile |
| Number of zero discharge days (annual) | Days above 10^th^, 30^th^, 50^th^, 70^th^ and 90^th^ percentiles. |
|  | Total for season* |
|  | **Average for season (Wet and Dry)*** |

**Supplemental table 3:** Description of river hydrograph data from the Tanami-Timor Sea Coast (TTS) utilised in study. Sources are as follows; GRDC is the Global Runoff Data Centre, BoM is the Bureau of Meteorology Water Data Online, NTG is the Northern Territory Government Aquatic Informatics site. Note, % complete includes filling minor gaps of 3 days or less using linear interpolation. Long term values of all water years with 90% or more complete records for mean flow (μQ), coefficient of variation of flow (CV_Q_), no flow days and the mean number of days per water year where flows were between historical 30^th^, 70^th^ and 90^th^ percentiles of flow.

| **Catchment** | **Station** | **Station number** | **Lat** | **Lon** | **Catchment area km^2^** | **Data from** | **Data**  **to** | **% Complete** | **Q**  **(ML/d)** | **CV_Q_ (%)** | **No flow days** | **Wet Season NDJFMA (ML/d)** | **Dry Season MJJASO (ML/d)** | **n years** | **Source** |
| --- | --- | --- | --- | --- | --- | --- | --- | --- | --- | --- | --- | --- | --- | --- | --- |
| Adelaide | Dirty Lagoon | 5708160 | -12.91 | 131.23 | 4325 | 1962 | 2021 | 68.1 | 4667.85 | 329.42 | 280 | 9742.23 | 121.31 | 59 | GRDC |
| Blyth | Mululu | G8240002 | -12.45 | 134.7 | 9087 | 1968 | 1986 | 91.8 | 1379.09 | 219.17 | 0 | 3958.01 | 222.40 | 19 | NTG |
| Daly | Mount Nancar | 5708145 | -13.83 | 130.74 | 47652 | 1967 | 2011 | 94.5 | 24584.48 | 200.90 | 0 | 43872.52 | 3025.72 | 46 | GRDC |
| E Alligator | 12^0^ 43’ S | 5708220 | -12.72 | 133.34 | 2384 | 1971 | 2014 | 76.8 | 3121.11 | 289.34 | 0 | 7411.38 | 192.67 | 43 | GRDC |
| Finniss | Hrs Gitchams | 5708170 | -12.97 | 130.76 | 1041 | 1969 | 2019 | 100 | 1541.36 | 281.77 | 1 | 2931.89 | 78.70 | 51 | GRDC |
| Fitzmaurice | Dakota Camp | 5708120 | -14.87 | 130.23 | 4920 | 1977 | 1980 | 75 | 2154.52 | 338.47 |  | 3435.37 | 203.35 | 3 | GRDC |
| Fitzroy | Willare | 5607025 | -17.74 | 123.65 | 91902 | 1998 | 2021 | 100 | 20277.77 | 258.86 | 25 | 39410.69 | 1234.70 | 24 | GRDC |
| Goomadeer | PL Tree DS Gorge | G8220217 | -12.38 | 133.57 | 5684 | 1971 | 1975 | 35.3 | 2288.23 | 133.10 |  | 7468.72 | 19.82 | 5 | NTG |
| Goyder | DS Old G Rd Xng 2 | G8250002 | -12.93 | 135.37 | 10391 | 1967 | 2002 | 54.2 | 2288.23 | 207.72 | 80 | 3798.70 | 391.24 | 36 | NTG |
| Isdell River | Dales Yard | 804001 | -17.01 | 125.43 | 19716 | 1967 | 2023 | 96.5 | 1040.66 | 379.76 | 39 | 2305.57 | 56.91 | 56 | BoM |
| Lennard | Mt Joseph | 803001 | -17.37 | 125.11 | 14747 | 1966 | 2023 | 98.4 | 734.05 | 478.59 | 172 | 1661.17 | 16.29 | 57 | BoM |
| Liverpool | DS Cuthb. Falls | G8230237 | -12.53 | 133.88 | 8941 | 1965 | 1986 | 95.5 | 1828.04 | 289.90 | 0 | 3106.29 | 142.75 | 22 | NTG |
| Mary | Mount Bundey | 5708185 | -12.91 | 131.65 | 5700 | 1956 | 2014 | 95.2 | 5709.09 | 288.36 | 51 | 11306.17 | 207.86 | 58 | GRDC |
| Sth Alligator | Hrs El Sherana | 5708190 | -13.52 | 132.52 | 1231 | 1958 | 2010 | 100 | 1438.97 | 289.78 | 14 | 2847.07 | 56.76 | 53 | GRDC |
| Victoria | Coolibah Hmstd | 5708110 | -15.55 | 130.96 | 44900 | 1953 | 2021 | 80.4 | 11938.96 | 339.89 | 77 | 25662.42 | 366.98 | 69 | GRDC |

**Supplemental table 4:** Description of river hydrograph data from the Carpentaria Coast (CC) utilised in study. Sources are as follows; GRDC is the Global Runoff Data Centre, BoM is the Bureau of Meteorology Water Data Online, NTG is the Northern Territory Government Aquatic Informatics site. Note, % complete includes filling minor gaps of 3 days or less using linear interpolation. Long term values of all water years with 90% or more complete records for mean flow (μQ), coefficient of variation of flow (CV_Q_), no flow days and the mean number of days per water year where flows were between historical 30^th^, 70^th^ and 90^th^ percentiles of flow.

| **_Catchment_** | **_Station_** | **_Station number_** | **_Lat_** | **_Lon_** | **_Catchment area km2_** | **_Data from_** | **_Data_**  **_to_** | **_% Complete_** | **_Q_**  **_(ML/d)_** | **_CVQ (%)_** | **_No flow days_** | **_Wet Season NDJFMA (ML/d)_** | **_Dry Season MJJASO (ML/d)_** | **_n_**  **_years_** | **_Source_** |
| --- | --- | --- | --- | --- | --- | --- | --- | --- | --- | --- | --- | --- | --- | --- | --- |
| _Archer_ | _Telegraph Rd_ | _5109240_ | _-13.42_ | _142.92_ | _2928_ | _1968_ | _2021_ | _98.1_ | _4576.29_ | _297.57_ | _56_ | _8559.40_ | _430.80_ | _53_ | _GRDC_ |
| _Calvert_ | _US Burke. Rd_ | _G9090249_ | _-17.21_ | _137.43_ | _10033_ | _1971_ | _1986_ | _75_ | _442.35_ | _370.06_ | _0_ | _1007.46_ | _15.38_ | _16_ | _NTG_ |
| _Embley_ | _Kurracoo Ck_ | _924001A_ | _-12.82_ | _142.18_ | _4692_ | _1971_ | _1986_ | _100_ | _728.99_ | _313.88_ | _149_ | _1448.66_ | _7.68_ | _15_ | _BoM_ |
| _Flinders_ | _Walkers Bend_ | _5109151_ | _-18.16_ | _140.86_ | _106300_ | _1969_ | _2021_ | _97.2_ | _9049.42_ | _352.36_ | _216_ | _17711.28_ | _163.19_ | _52_ | _GRDC_ |
| _Holroyd_ | _Ebagoola_ | _921001A_ | _-14.25_ | _143.17_ | _10218_ | _1970_ | _1988_ | _100_ | _513.79_ | _423.37_ | _191_ | _1066.50_ | _15.44_ | _20_ | _BoM_ |
| _Jardine_ | _Monument_ | _5109230_ | _-11.15_ | _142.35_ | _2421_ | _1978_ | _2021_ | _97.7_ | _5640.13_ | _101.46_ | _0_ | _7686.36_ | _3158.40_ | _43_ | _GRDC_ |
| _Leichhardt_ | _Floraville Hst_ | _5109210_ | _-18.23_ | _139.88_ | _23660_ | _1984_ | _2021_ | _92.2_ | _5354.88_ | _484.37_ | _178_ | _10783.21_ | _77.13_ | _37_ | _GRDC_ |
| _McArthur_ | _Borroloola Rd_ | _5709112_ | _-16.08_ | _136.32_ | _15700_ | _1975_ | _2021_ | _86.4_ | _4370.83_ | _313.92_ | _9_ | _9867.74_ | _344.38_ | _46_ | _GRDC_ |
| _Mitchell_ | _Koolatah Stn_ | _5109200_ | _-15.95_ | _142.38_ | _45872_ | _1972_ | _2012_ | _85.9_ | _22065_ | _259.28_ | _1_ | _43861.22_ | _1130.29_ | _41_ | _GRDC_ |
| _Robinson_ | _Robinson Hst_ | _G9080122_ | _-14.7_ | _134.42_ | _47400_ | _1969_ | _1986_ | _47_ | _1061.24_ | _383.18_ | _0_ | _2855.51_ | _89.75_ | _18_ | _NTG_ |
| _Roper_ | _Red Rock_ | _5709100_ | _-12.45_ | _142.64_ | _3290_ | _1966_ | _2021_ | _87.8_ | _8245.52_ | _270.01_ | _0_ | _18914.42_ | _371.41_ | _56_ | _GRDC_ |
| _Wenlock_ | _Hrs Moreton_ | _5109251_ | _-13.1_ | _142.94_ | _725_ | _1958_ | _2019_ | _100_ | _3836.77_ | _215.31_ | _5_ | _7161.66_ | _541.80_ | _62_ | _GRDC_ |

**Supplemental table 5:** Description of river hydrograph data from the North East Coast (NEC) utilised in study. Sources are as follows; GRDC is the Global Runoff Data Centre, BoM is the Bureau of Meteorology Water Data Online, NTG is the Northern Territory Government Aquatic Informatics site. Note, % complete includes filling minor gaps of 3 days or less using linear interpolation. Long term values of all water years with 90% or more complete records for mean flow (μQ), coefficient of variation of flow (CV_Q_), no flow days and the mean number of days per water year where flows were between historical 30^th^, 70^th^ and 90^th^ percentiles of flow.

| **Catchment** | **Station** | **Station number** | **Lat** | **Lon** | **Catchment area km^2^** | **Data from** | **Data**  **to** | **% Complete** | **Q**  **(ML/d)** | **CV_Q_ (%)** | **No flow days** | **Wet Season NDJFMA (ML/d)** | **Dry Season MJJASO (ML/d)** | **n**  **years** | **Source** |
| --- | --- | --- | --- | --- | --- | --- | --- | --- | --- | --- | --- | --- | --- | --- | --- |
| Barron | Myola | 5101100 | -16.8 | 145.61 | 1945 | 1915 | 2021 | 100 | 2129.13 | 273.49 | 0 | 3486.53 | 758.04 | 107 | GRDC |
| Black | Bruce Hwy | 5101170 | -19.24 | 146.63 | 255 | 1973 | 2007 | 99.9 | 212.42 | 674.06 | 190 | 402.85 | 28.17 | 35 | GRDC |
| Burdekin | Home Hill | 5101201 | -19.64 | 147.4 | 129939 | 1921 | 2001 | 100 | 11708 | 311.06 | 210 | 21597.13 | 1453.37 | 81 | GRDC |
| Daintree | Bairds | 5101080 | -16.18 | 145.28 | 907 | 1968 | 2008 | 98.2 | 2316.62 | 233.82 | 6 | 3487.55 | 1006.14 | 41 | GRDC |
| Endeavour | Flaggy | 107001B | -15.42 | 145.07 | 337 | 1958 | 1967 | 89.3 | 438.61 | 296.83 | 27 | 684.62 | 246.95 | 58 | QG |
| Herbert | Ingham | 5101162 | -18.63 | 146.14 | 8585 | 2009 | 2021 | 100 | 11201 | 250.60 | 0 | 18621.44 | 3237.78 | 12 | GRDC |
| Jeannie | Wakooka Rd | 5101060 | -14.76 | 144.86 | 323 | 1970 | 1988 | 85.5 | 437.55 | 344.94 | 128 | 862.21 | 16.45 | 19 | GRDC |
| Mulgrave | Gordonvale | 5101111 | -17.1 | 145.79 | 552 | 1916 | 1988 | 76 | 2190.76 | 211.03 | 0 | 3086.15 | 1175.52 | 74 | GRDC |
| N. Johnstone | Goondi | 5101117 | -14.92 | 144.21 | 12930 | 2005 | 2021 | 100 | 7478.30 | 263.23 | 0 | 15083.24 | 459.16 | 16 | GRDC |
| Normanby | Kalpowar Crs. | 5101071 | -12.66 | 143.05 | 1313 | 1970 | 2021 | 98.2 | 3642.39 | 259.19 | 3 | 6392.12 | 594.52 | 52 | GRDC |
| Pascoe | Garraway Ck | 5101020 | -17.53 | 145.97 | 936 | 1928 | 1968 | 100 | 4839.67 | 135.22 | 68 | 6431.22 | 3174.44 | 40 | GRDC |
| S. Johnstone | US Central Mill | 5101118 | -17.61 | 145.98 | 400 | 1916 | 2012 | 99.6 | 2234.85 | 159.77 | 0 | 3178.93 | 1284.11 | 97 | GRDC |
| Stewart | Telegraph Rd | 5101040 | -14.17 | 143.39 | 470 | 1970 | 2021 | 97.3 | 581.31 | 356.68 | 102 | 1103.01 | 30.47 | 52 | GRDC |
| Tully River | Euramo | 5101130 | -17.99 | 145.94 | 1450 | 1972 | 2021 | 99 | 8538.01 | 119.95 | 0 | 11169.40 | 5648.50 | 50 | GRDC |

**Supplemental table 6** Total species richness and species richness of trophic guilds by catchment including discharge metrics.

| **Catchment** | **Drainage basin** | **Total Species Richness** | **HD** | **OM** | **IN** | **PR** | **MaxQ** | **Q** | **CVQ** | **No Flow** | **Wet** | **Dry** | **n_years** |
| --- | --- | --- | --- | --- | --- | --- | --- | --- | --- | --- | --- | --- | --- |
| Archer-Watson | GoC | 46 | **2** | **13** | **20** | **11** | 135484.77 | 4387.59 | 289.28 | 56 | 8209.90 | 439.94 | 53 |
| Calvert | GoC | 31 | **3** | **7** | **12** | **9** | 16201.08 | 542.36 | 338.92 | 0 | 773.79 | 6.11 | 16 |
| Embley | GoC | 33 | **2** | **12** | **11** | **8** | 18705.14 | 690.07 | 301.07 | 149 | 1348.72 | 7.77 | 15 |
| Flinders-Norma | GoC | 46 | **5** | **10** | **18** | **13** | 172556.61 | 8586.59 | 348.77 | 216 | 16479.35 | 158.80 | 52 |
| Holroyd | GoC | 41 | **3** | **12** | **15** | **11** | 25301.53 | 522.60 | 416.68 | 191 | 964.19 | 15.57 | 20 |
| Jardine | GoC | 50 | **3** | **15** | **20** | **12** | 29359.60 | 5400.70 | 98.32 | 0 | 7358.65 | 3195.66 | 43 |
| McArthur | GoC | 39 | **4** | **10** | **16** | **9** | 131109.33 | 5187.41 | 278.39 | 9 | 8619.01 | 247.31 | 46 |
| Mitchell | GoC | 47 | **4** | **14** | **18** | **11** | 308687.97 | 18580.83 | 240.02 | 1 | 33821.97 | 997.91 | 41 |
| Leichhardt | GoC | 48 | **4** | **11** | **20** | **13** | 644246.80 | 5363.39 | 690.86 | 178 | 10783.21 | 77.13 | 37 |
| Robinson | GoC | 30 | **3** | **7** | **11** | **9** | 47185.73 | 2010.81 | 294.65 | 0 | 1817.79 | 22.15 | 18 |
| Roper | GoC | 46 | **4** | **12** | **19** | **11** | 128894.91 | 8994.97 | 260.10 | 0 | 14866.82 | 353.23 | 56 |
| Wenlock | GoC | 54 | **4** | **15** | **25** | **10** | 49729.54 | 3821.96 | 211.08 | 5 | 7022.18 | 546.26 | 62 |
| Barron | NEC | 52 | **6** | **14** | **23** | **9** | 265734.69 | 2129.13 | 273.50 | 0 | 3486.53 | 758.04 | 107 |
| Black | NEC | 29 | **4** | **8** | **10** | **7** | 43340.49 | 212.42 | 674.06 | 190 | 402.85 | 28.17 | 35 |
| Burdekin | NEC | 40 | **6** | **11** | **14** | **9** | 3355500.38 | 11708.17 | 311.06 | 210 | 21597.13 | 1453.37 | 81 |
| Daintree | NEC | 49 | **5** | **14** | **20** | **9** | 193708.37 | 2316.62 | 233.82 | 6 | 3487.55 | 1006.14 | 41 |
| Endeavour | NEC | 42 | **2** | **10** | **21** | **9** | 11097.94 | 455.99 | 283.74 | 0 | 604.92 | 229.11 | 58 |
| Herbert | NEC | 47 | **3** | **14** | **21** | **9** | 785468.02 | 11201.32 | 250.60 | 0 | 18621.44 | 3237.78 | 12 |
| Johnstone | NEC | 49 | **3** | **15** | **22** | **9** | 218165.44 | 4839.67 | 135.22 | 68 | 6431.22 | 3174.44 | 40 |
| Jeannie | NEC | 37 | **2** | **8** | **20** | **7** | 14457.04 | 457.12 | 342.95 | 128 | 729.89 | 13.90 | 19 |
| Mulgrave | NEC | 51 | **4** | **14** | **24** | **9** | 148176.00 | 2190.76 | 211.03 | 0 | 3086.15 | 1175.52 | 74 |
| Normanby | NEC | 39 | **2** | **8** | **19** | **10** | 61377.14 | 1790.56 | 356.37 | 0 | 3345.36 | 120.74 | 16 |
| Pascoe | NEC | 45 | **1** | **10** | **23** | **11** | 82972.63 | 3412.31 | 250.12 | 3 | 6187.82 | 600.17 | 52 |
| Stewart | NEC | 29 | **2** | **8** | **11** | **8** | 20248.80 | 553.07 | 347.19 | 102 | 1043.23 | 31.22 | 52 |
| Tully | NEC | 51 | **4** | **15** | **23** | **9** | 89916.57 | 8538.01 | 119.95 | 0 | 11169.40 | 5648.50 | 50 |
| Adelaide | TTS | 47 | **3** | **10** | **23** | **11** | 96076.86 | 5811.63 | 269.47 | 280 | 7501.37 | 99.08 | 59 |
| Blyth | TTS | 43 | **3** | **12** | **18** | **10** | 66333.71 | 2117.95 | 244.24 | 0 | 3496.01 | 210.22 | 19 |
| Daly | TTS | 51 | **5** | **15** | **19** | **12** | 230022.37 | 22841.59 | 194.10 | 0 | 40040.93 | 2829.68 | 46 |
| EastAlligator | TTS | 49 | **4** | **11** | **20** | **14** | 90252.64 | 3718.46 | 256.88 | 0 | 5452.55 | 161.07 | 43 |
| Finniss | TTS | 44 | **4** | **12** | **19** | **9** | 37810.63 | 1492.94 | 279.42 | 1 | 2897.51 | 79.34 | 51 |
| Fitzmaurice | TTS | 33 | **4** | **7** | **14** | **8** | 37670.40 | 1834.96 | 199.31 | 2 | 2415.88 | 111.10 | 3 |
| Fitzroy | TTS | 31 | **3** | **7** | **14** | **7** | 315468.30 | 19550.80 | 246.59 | 25 | 37507.00 | 1328.85 | 24 |
| Goomadeer | TTS | 41 | **3** | **10** | **18** | **10** | 43191.88 | 6737.16 | 120.42 | 0 | 3584.18 | 0.78 | 5 |
| Goyder | TTS | 46 | **4** | **13** | **19** | **10** | 35861.62 | 2088.59 | 195.42 | 80 | 2183.37 | 210.39 | 36 |
| Isdell | TTS | 27 | **3** | **8** | **11** | **5** | 46477.16 | 1133.85 | 366.84 | 39 | 2076.09 | 58.29 | 56 |
| Lennard | TTS | 23 | **3** | **5** | **9** | **6** | 51748.24 | 835.36 | 475.70 | 172 | 1535.68 | 16.42 | 57 |
| Liverpool | TTS | 42 | **3** | **12** | **18** | **9** | 57366.77 | 1563.84 | 264.61 | 0 | 2874.66 | 139.54 | 22 |
| Mary | TTS | 46 | **3** | **12** | **20** | **11** | 127800.42 | 5723.99 | 283.41 | 51 | 10703.18 | 189.28 | 58 |
| SouthAlligator | TTS | 55 | **5** | **14** | **24** | **12** | 42831.77 | 1446.03 | 289.95 | 14 | 2827.563 | 58.23161 | 53 |
| Victoria | TTS | 41 | **5** | **9** | **16** | **11** | 240745.97 | 14177.87 | 314.33 | 77 | 19477.13 | 194.42 | 69 |

**Supplemental table 7.** Model descriptions and results for GLMM analysis of Total Species richness across all discharge predictors. There was no variance in random effects therefore no Conditional R^2^.

| **Model** | **df** | **AIC** | **R^2^ Marginal** | **P value** | **Slope** | **SE** |
| --- | --- | --- | --- | --- | --- | --- |
| Total Richnes ~ Q + (1\| basin) | 36 | -2.83 | 0.18 | 0.01 | 0.08 | 0.03 |
| Total Richnes ~ Wet + (1\| basin) | 36 | -2.65 | 0.17 | 0.01 | 0.07 | 0.03 |
| Total Richnes ~ Dry + (1\| basin) | 36 | -4.10 | 0.22 | 0.002 | 0.05 | 0.02 |
| Total Richnes ~ CVQ + (1\| basin) | 36 | -5.35 | 0.18 | 0.01 | -0.24 | 0.08 |

**Supplemental table 8.** Pairwise contrast table for the four trophic guilds across four flow predictors.

| **contrast** | **estimate** | **SE** | **df** | **t ratio** | **P value** | **Flow Variable** |
| --- | --- | --- | --- | --- | --- | --- |
| Herbivore/Detritivore - Omnivore | 0.01 | 0.04 | 152 | 0.33 | 0.99 | Q_log |
| Herbivore/Detritivore - Invertivore | -0.01 | 0.04 | 152 | -0.21 | 0.99 | Q_log |
| Herbivore/Detritivore - Predator | 0.01 | 0.04 | 152 | 0.22 | 0.99 | Q_log |
| Omnivore - Invertivore | -0.02 | 0.04 | 152 | -0.53 | 0.99 | Q_log |
| Omnivore - Predator | -0.001 | 0.04 | 152 | -0.11 | 0.99 | Q_log |
| Invertivore - Predator | 0.025 | 0.04 | 152 | 0.42 | 0.97 | Q_log |
| Herbivore/Detritivore - Omnivore | 0.02 | 0.04 | 152 | 0.26 | 0.99 | Wet_log |
| Herbivore/Detritivore - Invertivore | -0.001 | 0.04 | 152 | -0.11 | 0.99 | Wet_log |
| Herbivore/Detritivore - Predator | 0.01 | 0.04 | 152 | 0.26 | 0.99 | Wet_log |
| Omnivore - Invertivore | -0.02 | 0.04 | 152 | -0.36 | 0.98 | Wet_log |
| Omnivore - Predator | 0.001 | 0.04 | 152 | 0.01 | 0.99 | Wet_log |
| Invertivore - Predator | 0.016 | 0.04 | 152 | 0.37 | 0.98 | Wet_log |
| Herbivore/Detritivore - Omnivore | -0.02 | 0.03 | 152 | -0.69 | 0.90 | Dry_log |
| Herbivore/Detritivore - Invertivore | -0.03 | 0.03 | 152 | -1.19 | 0.64 | Dry_log |
| Herbivore/Detritivore - Predator | 0.02 | 0.03 | 152 | 0.87 | 0.82 | Dry_log |
| Omnivore - Invertivore | -0.01 | 0.03 | 152 | -0.50 | 0.96 | Dry_log |
| Omnivore - Predator | 0.04 | 0.03 | 152 | 1.56 | 0.40 | Dry_log |
| Invertivore - Predator | 0.05 | 0.03 | 152 | 2.06 | 0.17 | Dry_log |
| Herbivore/Detritivore - Omnivore | 0.24 | 0.13 | 152 | 1.78 | 0.29 | CV_log |
| Herbivore/Detritivore - Invertivore | 0.29 | 0.13 | 152 | 2.20 | 0.13 | CV_log |
| Herbivore/Detritivore - Predator | 0.05 | 0.13 | 152 | 0.40 | 0.98 | CV_log |
| Omnivore - Invertivore | 0.06 | 0.13 | 152 | 0.41 | 0.98 | CV_log |
| Omnivore - Predator | -0.19 | 0.13 | 152 | -1.38 | 0.51 | CV_log |
| Invertivore - Predator | -0.24 | 0.13 | 152 | -1.79 | 0.28 | CV_log |


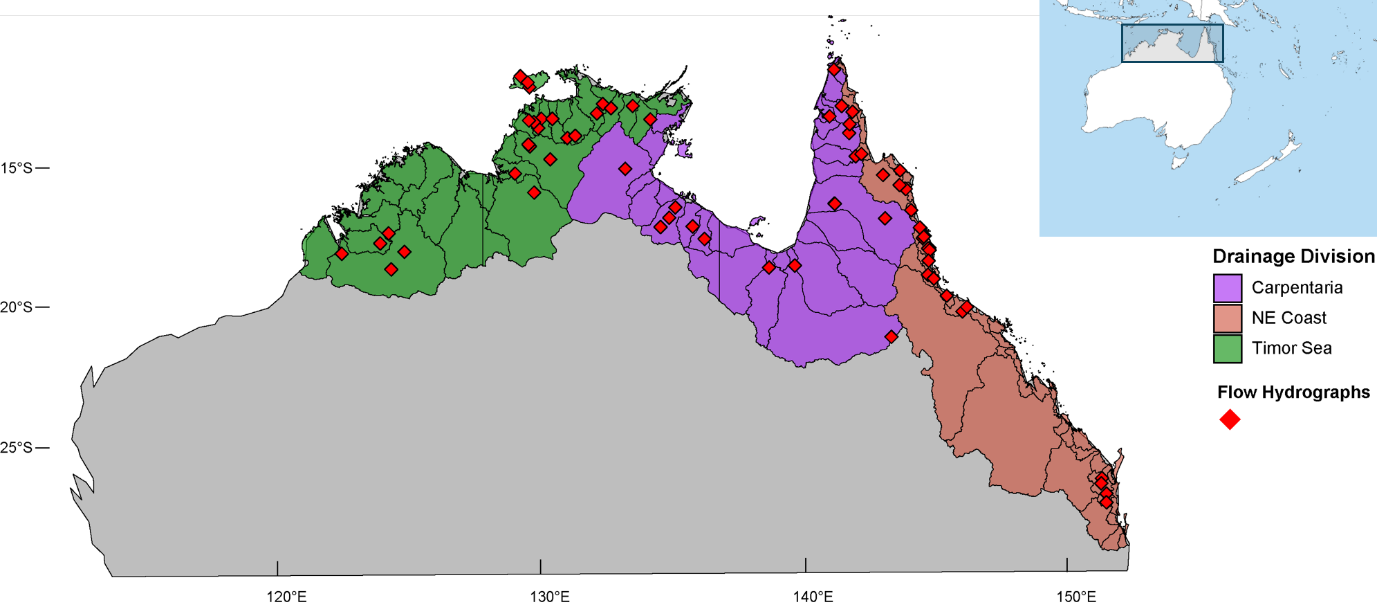


**Supplemental Figure 1.** Locations of the hydrologic gauging stations, drainage divisions and catchment boundaries used to assess fish species richness in tropical Australia.


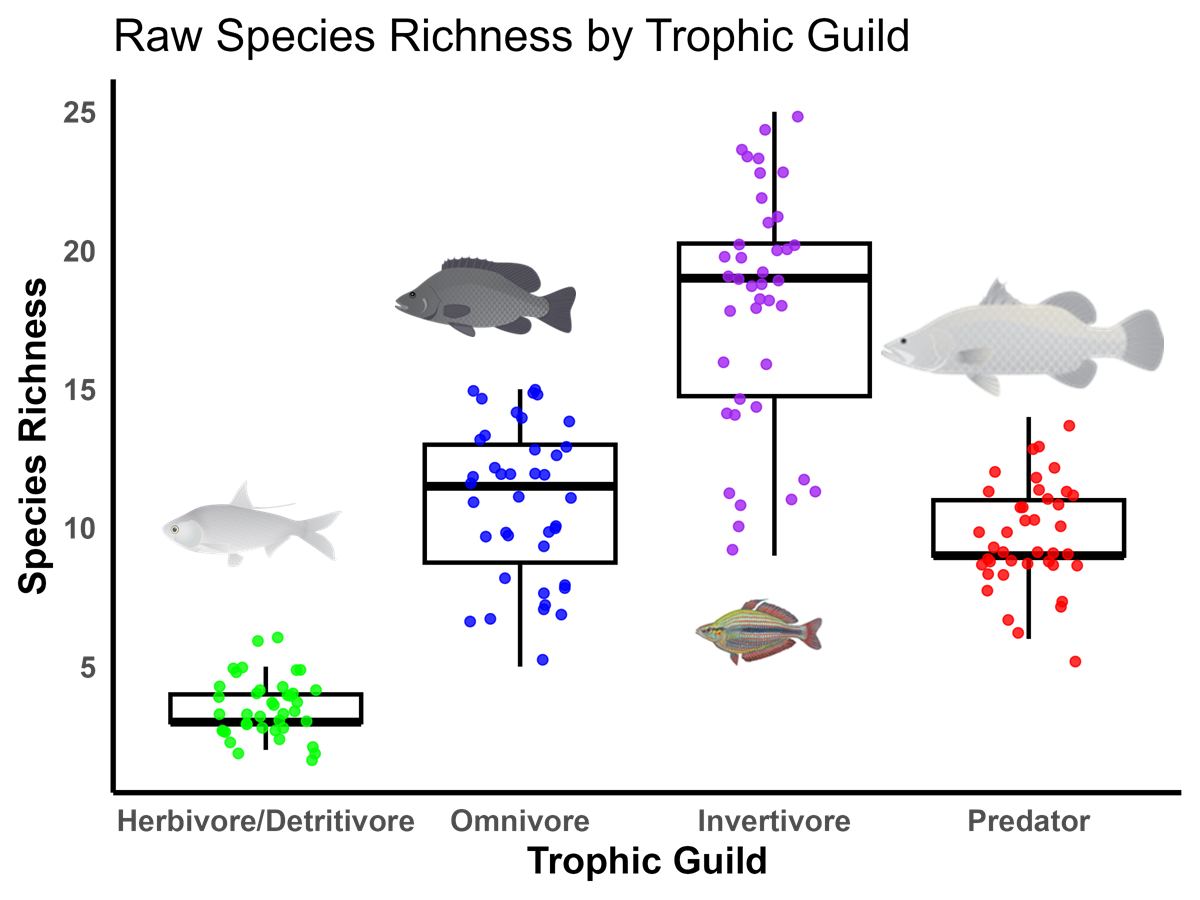


**Supplemental figure 2**. Box plot of trophic guild richness of freshwater fishes in tropical Australia, and coefficient estimates ± 95% confidence intervals.


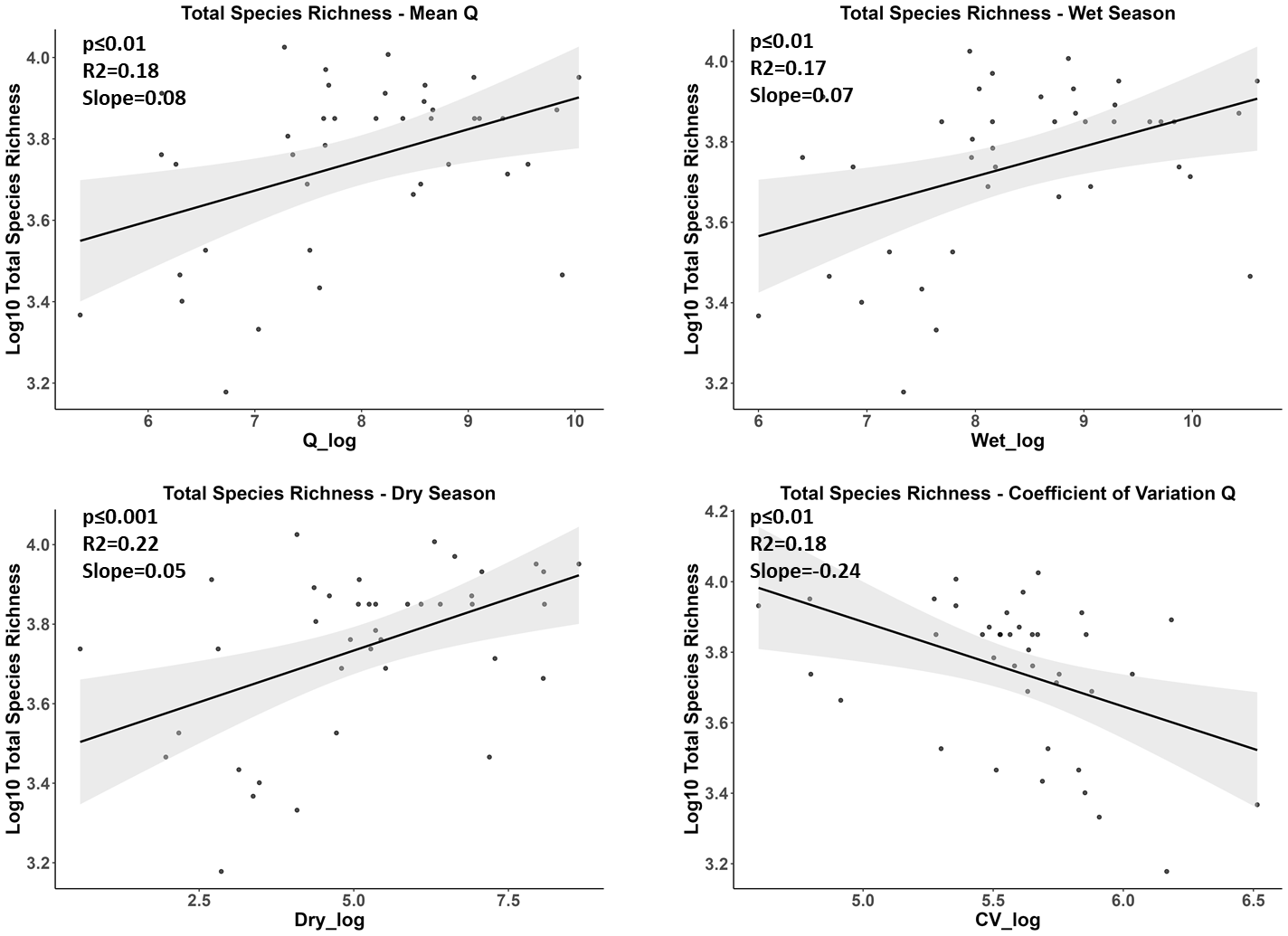


**Supplemental figure 3.** Fitted liner models slopes, including 95% confidence intervals in the shaded area, for total species richness of mean annual discharge Q (ML/d), Wet season discharge (ML/d), Dry season discharge (ML/d) and Coefficient of Variation for mean annual discharge (CVQ). All data was is log10 transformed. Each datapoint represents the number of species in a catchment.
